# Effect of comorbidities and risk conditions on death from COVID-19 in migrants in Mexico

**DOI:** 10.1186/s12939-021-01599-9

**Published:** 2021-12-18

**Authors:** Oscar A. Martínez-Martínez, Karla A. Valenzuela-Moreno, Brenda Coutiño

**Affiliations:** 1 Department of Social and Political Sciences, Universidad Iberoamericana, Prolongación Paseo de la Reforma 880, Álvaro Obregón, Lomas de Santa Fe, 01219 México, Mexico; 2 International Studies Department, Universidad Iberoamericana, Prolongación Paseo de la Reforma 880, Álvaro Obregón, Lomas de Santa Fe, 01219 México, Mexico

**Keywords:** Migrants, COVID-19, Comorbidities, Health, Mexico

## Abstract

**Background:**

Comorbidities increase the risk of death for patients with COVID-19, however, little is known about how it affects the prognosis of migrants who contract the virus. Therefore, this article aims to determine which comorbidities and risk conditions are associated with the probability of death among migrants infected with COVID-19 in Mexico.

**Methods:**

We use a sample of migrants with a positive diagnosis for COVID-19 (*N* = 2126) registered in the public database published in the National Epidemiological Surveillance System of the Mexican Ministry of Health; the technique used was a Probit regression.

**Results:**

The findings show that most of the comorbidities commonly associated with death from COVID-19 in the native-born population were actually not significant when present in migrants infected with COVID-19. Additionally, migrants have lower comorbidities than locals. The results further indicate that the factors related to the death of migrants infected with COVID-19 are: age, intubation, nationality group, pneumonia and the Health Care Management of Patients.

**Conclusions:**

In contrast to preceding studies with native-born populations with COVID-19, where pre-existing diseases aggravated the diagnosis of COVID-19 and sometimes led to death, in the case of migrants, only pneumonia was the significant comorbidity associated with mortality among migrants diagnosed with COVID-19.

**Supplementary Information:**

The online version contains supplementary material available at 10.1186/s12939-021-01599-9.

## Background

According to the World Health Organization (WHO), the coronaviruses cause respiratory infections, whose symptoms may range from a common cold to more serious syndromes, such as the Middle East Respiratory Syndrome (MERS) and the Severe Acute Respiratory Syndrome (SARS) [1]. The most recent coronavirus is the cause of the Corona Virus Disease, COVID-19. Since the first case of COVID-19 was registered in Wuhan China in December 2019, countries all over the world started preparing for this emergency by implementing various measures, such as lockdowns and social distancing [2]. Despite such efforts, as of September 2021, global deaths amounted to 4,571,729 [3].

Research has shown that there are comorbidities and risk conditions that increase the probability of death, like advanced age and coexistence of certain preexisting diseases [4]. In addition to medical conditions, there are social determinants of health that place certain populations at a higher risk [5]. These factors are determined by socio-economic conditions that reproduce inequalities, which have a detrimental effect on the health of individuals [6]. According to the WHO [1], some of these determinants are: income, education, structural conflict, working conditions, housing, social inclusion and discrimination. Food insecurity and its implications for migrants and racialized communities have also raised concerns during the pandemic [7].

These factors are usually present in populations of lower socio-economic status or minorities, namely: the poor, the elderly, indigenous communities, people of color and migrants [8]. Furthermore, it has been suggested that migration should be studied as a social determinant on its own, since it places individuals in uneven relationships with the state, particularly with public healthcare institutions [6] and oftentimes they face discrimination, violence and marginalization, leading to health disparities [9, 10].

Regardless of the pandemic, migrants -especially those with irregular status- and refugees face many risk conditions derived from the environment in origin, transit and destination countries [11]. The environment in these countries refers to “push” factors that stimulate emigration, such as low wages and unemployment, long working hours, poor social security [12] environmental degradation, gender violence, adverse political, and socio-economic conditions [13]. An additional risk condition is their ineligibility for healthcare at destination (except in case of emergency care) [14, 15]. Furthermore, people on the move are especially vulnerable to infectious diseases, due to high mobility, lack of sanitation and water systems, overcrowding living conditions, and lack of access to vaccination programs [14, 16].

In addition to the usual restrictions to access healthcare and risks associated with the migratory journey and settlement [17], these populations have been severely affected by some of the measures put into place worldwide in order to control the spread of COVID-19 [18]. Travel restrictions, border control reinforcement, the suspension of resettlement refugee programs and humanitarian assistance at borders, and the overcrowding at detention centers, have all exacerbated the health situation of migrants and refugees [11, 19]. Since the pandemic started, there has been an interest to determine which comorbidities tend to have negative effects in patients who contract SARS-CoV2. Cardiovascular diseases, diabetes, and hypertension complicate the health conditions of patients with COVID-19 [4, 20]. However, most of these studies involve the native-born population, while little is known about the effects of these preexisting diseases in migrants [4, 20]. Given this context, the research question we want to address is which comorbidities and risk conditions are associated with the probability of death among migrants who contract COVID-19.

### The changing migration context in Mexico

For the purpose of this study, migrants are considered people who move away from their countries of birth, across international borders, either temporarily or permanently, and for a variety of reasons [21]. In Latin America, migrants and refugees tend to be dispersed among the local population, with restricted access to social protection and healthcare [22], suffering from poverty, precarious living conditions, working under exploitation, and illegality, and oftentimes, unaware of their human rights [23]. All of these factors, along with discrimination, unemployment, mobility restrictions, xenophobia, and ineligibility for government aid, make them more vulnerable to COVID-19 [24].

In Latin America, little is known about how COVID-19 has affected migrants, and Mexico is no exception. The country has traditionally been studied by migration scholars, mainly due to its historical migration dynamics with the United States, and the fact that the US-Mexico migration flow constitutes the biggest migration corridor in the world [25]. Due to a shift in migration flows, Mexico has now become a country of destination, mainly for migrants coming from other countries in Latin America [26]. There are 1.2 million international migrants residing in Mexico, who constitute around 0.96% of the country’s total population. Most of the migrants come from the Americas, amounting to 317, 331, the majority born in the United States, followed by Latin American immigrants [27].

Recent shifts in mobility patterns have renewed interest in Mexico, now as a destination country. Some of these new flows are: migrant caravans from Central America; policy measures taken by the U.S. and deportations from overcrowded detention centers in the U.S., bound to Mexico and other countries [18, 28–30].

In 2020, most asylum applications in Mexico came from: 1. Honduras, 2. Haiti, 3. Cuba, 4. El Salvador, 5. Venezuela, 6. Guatemala, 7. Chile, 8. Nicaragua, 9. Colombia and 10. Brazil [31]. Therefore, although our study analyses migrants of different nationalities, we place special emphasis in Latin America and the Caribbean, since the number of migrants who need humanitarian assistance received by Mexico is on the rise. In addition, the countries of this region can be grouped based on certain migratory patterns. Migrants from Cuba and Haiti are more likely to eventually enter the US, when compared to migrants from other countries in Latin America [32], whereas Central Americans constitute one of the most vulnerable migrant groups in Mexico [30] and have been the most affected by the anti-immigrant backlash in the United States during Trump’s administration [33], and thus have been forced to wait in Mexico for longer periods [34]. Most migrants from these countries intend to continue their journey to the United States [35], so it is important to notice that it is not possible to assert which migrants will settle in Mexico.

Due to the COVID-19 pandemic, the United Nations (UN), the Office for the High Commissioner for Human Rights (OHCHR), the WHO, and the UNHCR, have recommended governments to adopt an inclusive approach against COVID-19, considering the precarious situation of migrants and refugees. These organizations have suggested to provide migrants indiscriminate access to preventive measures, testing and treatment [36].

The Federal Government has implemented an “Action Plan for Migrants’ Access to Healthcare during COVID-19”, which consists of a multi-level coordination to follow up positive cases and provide health assistance for migrants in shelters, mainly in the southern and northern border states [37]. Despite these measures, the fatality rate for migrants in Mexico is 6.05 and 29.18% of migrants tested positive for COVID-19 [38]. These results could be explained due to the fact that asylum seekers and refugees in the Mexican border states have an increased risk of contracting infectious respiratory diseases [39]; also, migration correlates positively with incidence of COVID-19 [40]. For this reason, the paper aims to determine which comorbidities and risk conditions are associated with the probability of death among migrants who contract COVID-19 in Mexico.

The relevance of knowing how comorbidities affect migrants in the Mexican context is two-fold: on the one hand, findings fill an existing gap in the literature, since there is a lack of research that points to the consequences of comorbidities on migrants who contract COVID-19; furthermore, Mexico is an understudied country in terms of its capacity for migrant settlement, since it has just recently been considered a country of destination [41]. Thus, knowing how comorbidities and risk conditions affect migrants in Mexico, becomes useful in order to inform public policy.

### Migrants´ health in times of COVID-19

The severity of respiratory viruses is associated with certain preexisting health conditions [42]. An increase in mortality was mostly associated with cardiovascular diseases [43], while advanced age patients (especially over 65) with comorbidities have more elevated admission rates into the Intensive Care Unit (ICU), as well as higher mortality rates [44]. A meta-analysis showed that the most common preexisting comorbidity in patients with COVID-19 is hypertension (15.8%), followed by cardiovascular and cerebrovascular diseases (11.7%), diabetes (9.4%), and co-existing infections (HIV and Hepatitis B).

Other comorbidities present in less proportion are malignancy, Chronic Obstructive Pulmonary Disease (COPD), and other respiratory system-related diseases, renal disorders and immunodeficiency states [45]. Despite the vulnerability of migrants, little research has been conducted regarding how COVID-19 has affected this group [46]. Some studies carried out in Europe at the beginning of the pandemic show that migrants who contract COVID-19 have lower or similar death rates when compared to non-migrants while hospitalization rates are higher for the latter [46, 47].

Migrants coming from low- or middle-income countries have higher mortality rates [48]. A study that compared characteristics of COVID-19 deceased patients by migration status, concluded that ischemic heart disease, hypertension, and autoimmune disease, were the most frequent comorbidities in deceased migrants, added to this, age was also a risk factor associated with death [49]. On the other hand, another research showed that the number of deaths correlated with comorbidities was lower for migrants; however, the positivity rates varied according to the origin of the migrants [50].

Singapore is one of the countries with lower case-fatality ratio in the world [51], because 88% of confirmed cases nationwide were concentrated among low-skilled migrant workers living in dormitories [52]. Ngiam et.al [53]. conducted a study to determine trends in hospitalized cases of these workers and they found that most migrants were asymptomatic and only 5.3% had a preexisting medical condition. Low mortality rates are partly explained by the migrants´ young age and fewer comorbidities, compared to local cases [53].

Research on migrant health has documented different health statuses and mortality rates between migrants and locals [54, 55]. Oftentimes, results show that migrants tend to have better health outcomes, less chronic diseases and lower mortality rates than the native-born at countries of destination [56, 57]. Even though migrants´ health advantage has received various explanations [58], the strongest hypothesis is that of the Healthy Migrant Effect (HIE).

The HIE states that migration is a self-selection process, in which healthier and younger people are more likely to migrate, since they need to be in good shape to endure the migration journey and go through the adaptation process in receiving societies [54, 59]. Adaptability and non-observable patterns, such as resilience and motivation, also explain the health selection processes [57]. It is also likely that migrants used to have better habits and healthier lifestyles at their countries of origin, compared to the native-born at their destinations [60].

It has been proved, however, that the longer migrants have resided in destination countries, the more likely it is for their health to deteriorate and ultimately converge with the health status of the native-born [61]. This could be explained by acculturation, increased exposure to low socioeconomic status, reduced access to health care, lack of social inclusion, and adoption of health-averse behaviors, such as smoking or poor diets [59, 62]. Another complementary hypothesis is the Salmon Effect or Salmon Bias [63, 64], which states that migrants tend to return to their countries of origin when their health is deteriorating; thus, only the healthiest migrants remain in destination countries.

## Methods

### Database

The information was obtained from an open access database published by the Ministry of Health of Mexico, which started registering cases in April 2020 with the first case of COVID-19 in the country. This site was launched as an integrated national surveillance system to collect information on all individuals with COVID-19 throughout the country. Each state is responsible for monitoring cases and reporting to the Federal Ministry of Health. Thus, the Ministry of Health gathers all the data through the National Epidemiological Surveillance System published by the General Direction of Epidemiology on a daily basis. These data set includes data on COVID-19 patients who were voluntarily tested for SARS-CoV-2, in both, public and private institutions (laboratories, primary care and hospitals). According to this criterion, the database records confirmed, negative, and suspected cases from different age groups, gender, ethnicity, native-born or migrant, and associated factors or diseases [65].

Until August 2021, 11,524 migrants were registered in the database, of which 8420 were negative cases, 875 suspected cases for which a final diagnosis was not obtained, and 2229 tested positive for COVID-19. We built our database from these numbers. In addition, considering 4.6% of the observations had missing values in three or more variables (that were not imputed) and these cases were excluded from the study; we decided to use the cases that had complete information in all the variables considered by the study; the database consisted of a final sample of 2126 migrant cases confirmed with COVID-19.

### Measure

The variables used in the study are found in Table 1. The death of the migrant by COVID-19 is determined by the Ministry of Health as part of the follow-up of deaths subject to epidemiological surveillance that is carried out on an ongoing basis [38].

**Table 1:** Description of Dependent and Independent Variables

Variables	Explanation	Values
***Dependent variable***		
Migrant’s death	Indicates whether or not the patient died from COVID-19.	1 = Death 0 = Otherwise
***Independent variables***		
Nationality group	Identifies the migrant’s region of origin	1 = Central America 2 = South America 3 = Caribbean region 4 = Other
Hypertension	Identifies whether the patient has a diagnosis of hypertension.	1 = Has hypertension 0 = Otherwise
Cardiovascular	Identifies whether the patient has a diagnosis of cardiovascular disease.	1 = Cardiovascular conditions 0 = Otherwise
Obesity	Identifies whether the patient has a diagnosis of obesity.	1 = If obese 0 = Otherwise
Diabetes	Identifies whether the patient has a diagnosis of diabetes.	1 = Has diabetes 0 = Otherwise
Immunosuppression	Identifies whether the patient is immunosuppressed.	1 = Immunosuppressed 0 = Otherwise
Pneumonia	Identifies whether the patient was diagnosed with pneumonia.	1 = If pneumonia is present 0 = Otherwise
Asthma	Identifies whether the patient has a diagnosis of asthma.	1 = Holds asthma 0 = Otherwise
COPD	Identifies whether the patient has a diagnosis of COPD	1 = With a diagnosis of COPD 0 = Otherwise
Sex	Biological distinction that classifies people into men or women.	1 = Female 0 = Man
Age	Identifies the patient’s age	Continuous variable
Smoking	Identifies if the patient has a smoking habit	1 = Has a smoking habit 0 = Otherwise
Days elapsed	It is the difference between the date of appearance of the symptoms and the confirmation of having COVID-19 through a test.	Continuous variable
HCMP	Identifies the type of care the patient received on the medical unit. It is labeled as outpatient if he/she returned home or is labeled as hospitalized if he/she was admitted to the hospital.	1 = If the case is ambulatory 0 = If the patient is hospitalized
Intensive Care Unit	Identifies whether the patient required admission to an Intensive Care Unit.	1 = Admitted to ICU 0 = Otherwise
Intubated	Identifies if the patient required intubation or not.	1 = Intubated patient 0 = Otherwise

Source. Author’s elaboration

Independent variables considered comorbidities, risk factors and nationalities. In the first group, we include conditions such as obesity, cardiovascular diseases, hypertension, diabetes, immunosuppression, pneumonia, asthma, and COPD. These aspects have shown a strong association with the probability of death from COVID-19 [61, 63, 64]. The risk conditions included in the study are age, sex, and smoking, based also on different previous researches that point to their association with death from the virus [4, 20, 39, 45]. In addition, we added clinical variables on the procedures that migrants underwent during the course of the disease, since it has been documented that the treatment of manifestations derived from the immune response triggered by the SARS-CoV-2 coronavirus is associated with the development of more severe symptoms of COVID-19, which can result in risk conditions for migrants [66]. The clinical variables are Health Care Management of Patients (HCMP), days elapsed, and whether the patient required admission to ICU or intubation.

The nationalities present in the database were grouped into four blocks, considering certain shared migratory patterns, as shown by various investigations previously described [30–35, 44]:

- Central Americans: Belize, Costa Rica, El Salvador, Guatemala, Honduras, and Nicaragua
- South Americans: Argentina, Brazil, Chile, Colombia, Ecuador, Peru, Uruguay, and Venezuela
- Caribbean: Cuba, Dominican Republic, Haiti, and Jamaica
- Others: United States, Spain, Germany, France, Italy, China, Canada, and others

### Procedures and data analysis

To evaluate the likelihood of death for COVID-19 among migrants, a probit regression analysis was performed [64], through the equation:

$$\mathit{\Pr}\ \left(y=1|x\right)=\varnothing (xb)$$

Where ∅ the standard cumulative normal probability distribution and *xb* is called the probit index. Since *xb* has a normal distribution, the interpretation of a probit coefficient *b*, is that an additional unit of the predictor leads to an increase of *b* standard deviations of the probit index. Then, the log likelihood function for the probit is:

$$\ln L=\sum {w}_j\ln \varnothing \left({x}_jb\right)+\sum {w}_j\mathit{\ln}\ \left[1-\varnothing \left({x}_jb\right)\right]$$

where *w*_*j*_ are weights.

The probit model uses the normal cumulative probability distribution function:

$$F(Z)={\int}_{-\infty}^{Z_0}\frac{1}{\sqrt{2\pi \sigma}}\ {e^{-}}^{{\left(z-{\mu}_2\right)}^2/\left(z-{\mu}_2\right)}$$

The model estimates the influence that the variables have on the probability of dying because of COVID-19 when considering the values of the explanatory variables. This latter fact is calculated by the partial derivative $\partial {P}_i\left/ \partial {X}_{ij}\right.=f\left({X}_i^{\prime}\beta \right){\beta}_j$ where *f*(…) is the probability distribution function of a variable with a standard normal distribution, so that $\partial {P}_i\left/ \partial {X}_{ij}\right.$ it also depends on the values taken by the independent variables (*X*) . As a measure of goodness-of-fit for this model, the Mc Fadden *R*^2^ called pseudo R-squared is considered.

$${R}^2=1-\frac{Ln\ {L}_0}{ Ln L\left({\beta}_{mv}\right)}$$

Where *Ln L*_0_ is the logarithm of the likelihood function under the constraint that all coefficients, except the constant, are zeros, and *Ln L* (*β*_*MV*_) is the logarithm of the unconstrained maximum likelihood function. Thus, in a probit model the evaluation of the coefficients is the same as for an OLS model.

According to the goodness-of-fit test for the probit model, pseudo- R2 value was very similar to McFadden’s R2 value, which was found as 0.5863 (see Table 2). In addition, very low value of Akaike Information Criteria (AIC) and the negative value of Bayesian Information Criteria (BIC) ensure that the goodness-of-fit of the probit model was satisfactory.

**Table 2:** Characteristics of the migrant population with COVID-19 (*N* = 2126)

**Dichotomous Variables**	***Yes***	***No***
Migrant death	3.7%	96.3%
Hypertension	10.1%	89.9%
Cardiovascular	1.3%	98.7%
Obesity	7.9%	92.1%
Diabetes	5.3%	94.7%
Immunosuppression	0.9%	99.1%
Pneumonia	11.7%	88.3%
Asthma	3.5%	96.5%
COPD	0.8%	99.2%
Smoking	7.5%	92.5%
ICU ^a^	19.4%	80.6%
Intubated ^a^	13.4%	86.6%
**Categorical Variables**	**Values**	**%**
Nationality groups	Central America	31.8%
	South America	32.4%
	Caribbean Region	6.9%
	Other countries	28.9%
Days elapsed	Mean	3.8
HCMP	Hospitalized	15.1%
	Ambulatory	84.9%
Sex	Male	58.9%
	Female	41.1%
**Interval Variables**		
Age	Mean	36.65
	Range	6–74

^a^ Percentage as proportion of hospitalized patients

Source. Author’s elaboration, from the MMH (2021c)

Multicollinearity was calculated by a maximum variance inflation factor (VIF), which in almost all the variables was within the acceptance threshold, with VIF’s less than 10 [67]. In the cases that exceeded this threshold, these were categorical variables that did not represent an association with other variables, and instead were associated with a low proportion of cases. For this reason, the result suggests that the explanatory variables specified in the model do not cluster together or exhibited multi-collinearity tendencies [68].

## Results

Table 2 shows the main characteristics of migrants with COVID-19. The primary region of origin was South America (32.4%), followed by Central America (31.8%). Concerning sociodemographic traits, 58.9% are men and 41.1% women and the mean age was 36.65 years. People reported smoking represents 7.5%. The proportions of comorbidities among migrants were around 5 to 12%, the highest was pneumonia (11.7%), hypertension (10.1%), and obesity (7.9), most of these were lower than in the native-born (see supplementary information). The variable days elapsed had a mean of 3.8 days, between the appearance of symptoms and the confirmation of having COVID-19 through a clinical test. The patients admitted to the hospital were 15.1 and 84.9% cases were ambulatory. Among the hospitalized patients, we found similar rates between ICU (19.4%) and intubation (13.4%). The percentage of dead migrants was 3.7%, of which 93.67% died in hospitals and 6.33% in a different place (home, shelters, among others) and these are people whose cases were treated as ambulatory.

The results of the marginal effects for the probability of death from COVID-19 in the migrant population with positive test for this disease, indicates that five factors had a significant relationship with the dependent variable: nationality groups (other), pneumonia, age, HCMP, and being intubated (see Table 3). Regarding the first, the marginal effect values show that the probability of dying is 0.015 percentage points (pp) higher for the group “Others”, compared to the migrants from South America.

**Table 3:** Probit results and Marginal effects of the explanatory variables on the probability of death from COVID-19

	Probit regression			Marginal effects	
	Coef	Std Error	*P* value	dy/dx	Std Error
Nationality groups (ref: South America)					
Central America	0.256	0.246	0.295	0.008	0.008
Caribbean Region	0.015	0.451	0.973	0.000	0.013
Other	0.446	0.225	0.047**	0.015	0.007
Hypertension	0.123	0.212	0.562	0.004	0.007
Cardiovascular	0.396	0.462	0.392	0.014	0.016
Obesity	0.320	0.214	0.130	0.011	0.007
Diabetes	0.092	0.233	0.691	0.003	0.008
Immunosuppression	0.534	0.578	0.355	0.019	0.020
Pneumonia	0.922	0.219	0.001***	0.032	0.007
Asthma	−0.135	0.427	0.752	−0.004	0.015
COPD	−0.030	0.495	0.952	−0.001	0.017
Sex	−0.285	0.188	0.130	−0.009	0.006
Age	0.021	0.005	0.001***	0.000	0.000
Smoking	−0.300	0.345	0.385	−0.010	0.012
Days elapsed	0.032	0.022	0.143	0.001	0.000
HCMP	− 142.740	27.840	0.001***	−0.881	0.003
ICU	−0.389	0.263	0.139	−0.014	0.009
Intubated	1.850	0.272	0.001***	0.064	0.009
Goodness of fit statistics					
LR chi2(18)	= 395.930				
Prob > chi2	= 0.000				
Pseudo R2	= 0.586				

***0.001 significance level

** 0.05 significance level

Source. Author’s elaboration, from the MMH (2021c)

The probability of death from COVID-19 for migrant people is increased by 0.32 pp. if the patient had a previous diagnosis of pneumonia. In relation to age, the probability of death from COVID-19 increased by 0.001 pp. for each additional year of age of the migrant.

The HCMP variable shows that the probability of death from COVID-19 decreases by 8.8 pp. if the patient received outpatient medical care. Finally, the probability of death from COVID-19 for migrant people is increased by 0.6 pp. if the patient is intubated.

## Discussion

International evidence shows that the population with the highest risk of contracting SARS-CoV2 and developing complications are those who have comorbidities and risk conditions [4, 20, 69]; the World Health Organization identified 14 high-risk underlying health conditions in COVID-19 patients [70, 71]. Comorbidities have different implications, since it has been found that patients admitted into intensive care have a greater number of comorbidities, compared to those who do not enter this area [20], in addition to presenting fewer encouraging results in their COVID-19 diagnoses [72] and a higher probability of developing severe symptoms [73].

Studies have repeatedly shown that comorbidities and risk conditions, such as cardiovascular diseases, diabetes, hypertension, and COPD [4, 69] are significantly associated with an increased risk of death related to SARS-CoV2 [74]. A relevant aspect is that most of these studies were carried out on people who are already settled in a given context, which does not necessarily include migrants. Our findings for migrants with COVID-19 show a somewhat different behavior: with the exception of pneumonia, which was the only comorbidity related to death from COVID-19, most of the comorbidities that are commonly associated with death from COVID-19 [44, 75] were not significant for migrants, as was found in some exploratory studies, which also found that only a low percentage of migrants had comorbidities [50, 53], leading to lower mortality rates compared to locals [46, 47].

In the context of the COVID-19 pandemic, our results could be explained in part by the HIE, because migrants who contract COVID-19 have fewer comorbidities than natives (see supplementary information). Furthermore, except for pneumonia, it seems that comorbidities affect them less in the probability of death from COVID-19. This could be explained, firstly, because migration is a process of self-selection [60, 76], so those who decide to migrate are usually the healthiest among their communities [54, 59], which is consistent with several studies [56, 57] that have shown that migrants have fewer comorbidities, as well as better health outcomes and lower mortality rates, compared to natives in destination countries. Second, the Salmon effect is also useful to explain our results, since it points to the likelihood of migrants returning to their home countries once they find out they suffer from deteriorating health conditions [63, 64], so this could be the case of migrants who were aware of the high risk of contagion and decided to return to their countries of origin. A third explanation, which renders the HIE relevant for our study, is the fact that, although migrants who contracted COVID-19 may have faced a series of factors such as border restrictions, long waiting periods and overcrowding in detention centers [17, 18], they still have lower probability of death related to comorbidities when compared to locals.

With respect to the mortality rates for infectious diseases in migrants, previous studies have shown that it is higher in this population as a result of high prevalence in origin countries and lack of access to healthcare at destinations [77]. Specifically, pneumonia is one of the most prevalent infectious disease in migrants and refugees, mainly due to the lack of adequate living conditions and appropriate distancing [78, 79]. Based on this information, it is expected that pneumonia would affect the health outcomes of migrants who contract COVID-19.

The findings show that the group of countries that make up “Other” are more vulnerable to death, especially when compared to South Americans. This is consistent with the fact that this group has the highest number of comorbidities and highest percentages in pre-existing diseases, which generate a higher probability of death from COVID-19 [20, 72, 73]. For example, the total number of migrants who reported diabetes (43.75%) belongs to this group, the same happens in the case of COPD (64.71%), obesity (35.12%) and pneumonia (36.29%); the latter was the only comorbidity which significantly increased the probability of death in migrants.

The results for this group could also be influenced by its composition, since 70.19% are Europeans, Americans, and Canadians; even though we do not know their time of residence in Mexico (being one of limitations of the database), they have probably been in the country longer that Latin Americans and Caribbean, since immigration from these latitudes has only recently been on the rise [31]. Following the HIE, the longer migrants have resided in the country, the more likely their health becomes similar to that of locals, as other studies have shown [61, 62].

In further consideration of the composition of the group “Other”, it is relevant that 43.97% is American. Research about American population residing in Mexico shows that nine out of ten decide to settle in the country based on family reunification [80], which may be an indicator that this group is mainly composed of second-generation migrants [81], that is, American-born persons of Mexican origin. Following this argument, our results are explained due to the fact that a large number of the persons that make up the group “Other”, already shared similarities with the Mexican-born, such as risk behavior patterns and lifestyles, hence, they had to go through a milder acculturation process (if at all).

In relation to Latin American and Caribbean migrants, all have the same probability of death. Despite the fact that this group endures more risks associated with the migratory journey [17], accommodation in transit and destination countries, they seem to be healthier than the nationalities discussed above. This could also be explained by the HIE, so only the healthiest among these nationalities [60, 76] were able to migrate and make it to Mexico, considering the difficult circumstances the journey entails.

For all groups, age was a risk condition, like other studies for locals and migrants show [44, 53], it correlates positively with the probability of death. The problem worsens with increasing age, as the probability of death increases with each additional year of age, as has been found in other studies [4, 44, 49].

The findings in this study show that migrants with COVID-19 have low prevalence of preexisting diseases. This could be explained due to the migrants’ younger age and fewer comorbidities than locals (see Table 2), which are in accordance with the HIE. According to our results, sex does not influence the probability of death in migrants with COVID-19 as it does in the native-born population, where men have higher mortality rates [82, 83]. With respect to smoking, this risk condition has shown ambiguous results in previous research, so it is even more uncertain how it plays out with the probability of death in patients with COVID-19 [84]. Further research is needed in order to determine the impact of sex and smoking in migrant populations who contracted this disease.

## Conclusions

Despite positive results for immigrants, this population group do pose certain challenges to public policies in the countries of destination, so specific measures need to be taken in order to protect migrant communities not only from dying, but also from contracting the virus [14]. This observation is particularly relevant for migrants who contract pneumonia, since it was the only comorbidity that correlated positively with the probability of death from COVID-19. Further measures need to be taken in order to prevent and control pneumonia in migrant communities, especially among Latin American and Caribbean Migrants (second group with the highest percentage of this disease), since the complexity of their migration journey places them at a higher risk. In the case of the nationalities grouped as “other”, more research is needed in order to determine the causes that led to them contracting pneumonia, so preventive measures can also be implemented for this group.

There is little literature about Mexico as a settlement country [41] so the conclusions presented in the study may be relevant to start creating disaggregated data on immigrants´ health -especially during the pandemic- in order to provide better services for migrants who settle in the country.

It is important to recognize some limitations in our study. First, when using data from the National Epidemiological System, these are collected with samples obtained by people who are tested for COVID-19 in laboratories and public or private hospitals, but not of those who did not get tested. In general, migrants report lower use of health services, compared to the native-born [85], so it is possible that migrants with an initially mild COVID case decided to remain home or at the shelters, and thus, it is impossible to determine whether they had any comorbidities or risk conditions that led to their deceases. Similarly, since there is no follow-up of all the positive cases that were not admitted to the hospital when they first arrived and were classified as ambulatory cases, it is not possible to determine whether more migrants died than those reported by the National Epidemiological System (see Table 2).

Second, the database did not disaggregate the type of migrant (e.g., first or second generation), so it was not possible to include any of this information in the study. Therefore, there is no information on the migration status of the sample, nor any indication that may shed light to whether they were in transit, live in shelters, or time of residence in Mexico. This information is important because it may help determine future public policies targeting a certain migrant profile that may be more prone to contracting COVID-19 and dying from it.

Finally, our study is consistent with other research [47, 49, 51] carried out in different contexts, in which comorbidities are less frequent in migrants and their prognosis after contracting COVID-19 is more optimistic when compared to non-migrants.

## Supplementary Information

**Additional file 1 Table**.**4** People with COVID-19.

## Data Availability

The datasets generated and/or analyzed during the current study are available in the Mexican Ministry of Health repository: https://www.gob.mx/salud/documentos/datos-abierto-152127

## Abbreviations

COPD: Chronic Obstructive Pulmonary Disease
HIE: Healthy Migrant Effect
ICU: Intensive Care Unit
MERS: Middle East Respiratory Syndrome
MMH: Mexican Ministry of Health
OHCHR: Office for the High Commissioner for Human Rights
PP: Percentage Points
SARS: Severe Acute Respiratory Syndrome
UN: United Nations
UNHCR: United Nations High Commissioner for Refugees
WHO: World Health Organization
