# Supplementary material for: Effect of comorbidities and risk conditions on death from COVID-19 in migrants in Mexico
Source: Int J Equity Health. 2021 Dec 18;20:257. doi: 10.1186/s12939-021-01599-9 (PMC8683816; doi:10.1186/s12939-021-01599-9)
Supplement: Supplementary file 1 — Additional file 1 Table.4 People with COVID-19. [file 12939_2021_1599_MOESM1_ESM.docx]

**Supplementary Information**

**Table. 4 People with COVID-19**

| **Comorbidities** | **Migrants**  **N=2,126** | **Native-born**  **N= 2,831,978** | **P-Value** |
| --- | --- | --- | --- |
| Hypertension | 10.1% | 15.69% | 0.001*** |
| Cardiovascular | 1.3% | 1.38% | 0.328 |
| Obesity | 7.9% | 13.33% | 0.001*** |
| Diabetes | 5.3% | 12.08% | 0.001*** |
| Immunosuppression | 0.9% | 0.74% | 0.224 |
| Pneumonia | 11.7% | 12.86% | 0.0433** |
| Asthma | 3.5% | 2.09% | 0.001*** |
| COPD | 0.8% | 0.99% | 0.161 |

***0.001 significance level

** 0.05 significance level

Source. Author’s elaboration, from the MMH (2021c).
